# Exploration of Textile–Silicone Composites and Materials for Personal Impact-Resistant Protection

**DOI:** 10.3390/ma17061439

**Published:** 2024-03-21

**Authors:** Mei-Ki Chan, Pui-Ling Li, Kit-Lun Yick, Joanne Yip, Sun-Pui Ng

**Affiliations:** 1 School of Fashion and Textiles, The Hong Kong Polytechnic University, Hong Kong, China; meiki-0125.chan@connect.polyu.hk (M.-K.C.); puiling-sabrina.li@polyu.edu.hk (P.-L.L.); tcjyip@polyu.edu.hk (J.Y.); 2 School of Professional Education and Executive Development, The Hong Kong Polytechnic University, Hung Hom, Kowloon, Hong Kong, China; zerance.ng@cpce-polyu.edu.hk

**Keywords:** textile–silicone composites, physical and mechanical properties, inlaid knitted spacer fabric, force protection, impact resistance

## Abstract

Conventional cushioning materials such as silicone sheets which have been recommended for resisting impact generally cause discomfort to the wearer from heat and perspiration. With the increasing need for personal protective equipment, textile–silicone composite structures are proposed in this study to reduce acute impact and moisture while enhancing thermal comfort. The influence of the composite structure and thickness on the mechanical and thermal properties of textile–silicone materials are systematically investigated. The results show that an additional knitted powernet fabric as a composite material can significantly improve the tensile properties of silicone rubber by up to 315%. However, only a slight improvement is found in the thermal conductivity (up to 16%), compression elasticity (up to 18%) and force reduction performance (up to 3.6%). As compared to inlaid spacer fabric, which has also been used for cushioning and preserving thermal comfort, the textile–silicone composites have higher tensile and compression elasticity, exhibit force reduction with the largest difference of 43% and are more thermally conductive, with increases more than 38%. The findings of this study introduced a cost-effective new silicone–textile composite for optimal impact protection and wear comfort for protective applications.

## 1. Introduction

Personal protective equipment (PPE), such as helmets, goggles, gloves and body suits, is crucial equipment that ensures health and safety to minimize hazards in workplace environments. According to the US Occupational Safety and Health Administration (OSHA) [1], PPE is designed to protect workers from serious workplace injuries or illnesses that result from contact with chemical, radiological, physical, or other workplace hazards. Protective apparel products are commonly used to protect workers from crushing or puncture injuries, and even from falling or flying objects in the construction and manufacturing industries. A prolonged exposure of the hand to the vibration of electrical and pneumatic-powered hand tools may cause damage to the vascular, neurological and musculoskeletal systems [2,3]. Anti-vibration gloves made of rubber, foam, or gel materials are designed to protect the hands from excessive vibration [4]. Recently, the safety and overall well-being of athletes have also been topics of concern [5] with the emergence of new technologies in sports applications to facilitate faster speed, more power and enhanced performance. This has led to the increasing requirement for appropriate materials and structural components to design sports protective equipment that would ensure sports integrity and protection [6]. To prevent the risk of head injuries, for example, protective helmets are generally constructed with a rigid outer shell that absorbs the initial impact energy and spreads the impact over a larger area. The impact energy is subsequently delocalized and transferred to the liner which is generally made of foam materials for effective shock attenuation. In the field of rehabilitation, prosthetic liners are typically made of thermoplastic elastomer or silicone materials to create a snug fit between the limb and prosthetic, absorb shock and reduce discomfort during movement. They offer intact contact with the residual limb, so its quality is a key factor which affects wear comfort and the ability to control the device. A well-fitting prosthetic liner enables uniform pressure distribution over the residual limb and reduces the effects of high-impact forces and the associated residual-limb pain during walking [7,8,9].

However, additional cushioning materials such as silicone sheets or hydrogels, have poor heat and moisture transport properties. This can lead to excessive sweating, pruritus and even skin breakdown [9,10]. In the application of prostheses and orthoses, cushioning materials that offer better porosity and breathability are preferred to reduce moisture and heat generation for improved wear comfort in the intact contact areas. A layer of textile fabric or liner is typically embedded into the cushioning materials to increase heat dissipation, improve wear comfort and reduce shear forces between the skin and liner. Advances in textile technologies, such as the sol-gel technique, can readily enhance textile surfaces’ repellence [11], while the application of a coated fabric on shape memory polyurethane can significantly alter water vapour permeability [12]. Despite the excellent tensile recovery of silicone elastomers, they exhibit poor tear and tensile strengths, along with reduced durability. An additional layer of fabric material can therefore also enhance the wear resistance and tear strength and dry feeling of silicone elastomers, thus broadening the applications for silicone rubber composites [13,14]. Chow et al. [15] developed a customized approach to fabricate a silicone composite which is additively manufactured onto fabric directly for hypertrophic scar treatment. Regrettably, most research on fabric-silicone composites has primarily focused on bonding performance and conductivity in sensor systems [14,16]. There have been very few studies that investigate the structural design, mechanical behaviour and impact-resistant performance of textile–silicone materials for effective protection of the human body.

Recently, the use of spacer fabric, which is composed of two outer layers tightly connected by a middle layer of spacer filaments, has attracted significant attention. This three-dimensional (3D) sandwich structure endows spacer fabric with excellent pressure relief and cushioning properties. Previous researchers have utilized spacer fabrics as protective padding to shield and support the body from injury in applications such as protective clothing [17], insoles [18], wound dressings [19] and protective helmets [20]. To further enhance the cushioning effect, researchers have proposed the use of silicone tubes as the inlay material. The tubes are inserted into the spacer structure during knitting to provide additional support with increased compressive resistance. Compared to conventional cushioning insole materials such as polyurethane and leather, textile-fabricated insoles made of a spacer structure with an inlaid silicone tube significantly reduce the relative humidity of the skin of the sole and heel, thus preserving a comfortable skin condition and a favourable sensation of thermal comfort during activities [18].

Since the cushioning performance and thermal behaviour of textile–silicone composites have been largely neglected, the purpose of this study is to provide a better understanding of the mechanical behaviour and thermal comfort properties of textile–silicone composites as compared to the traditional silicone elastomer and knitted spacer structure with a silicone tube inlay. We hypothesize that the textile–silicone composite structure will result in higher tensile elasticity and compression elasticity as compared to silicone rubber. The additional layer of textile material may also improve the thermal conductivity and water vapour permeability, which can transport sweat away from the skin and are therefore desirable to enhance the thermal comfort of wearers. The findings of this study will greatly contribute toward the fabrication of PPE and wearable cushioning products and widen the scope of the applications of textile-composite structures for improving the performance of protective wearables.

## 2. Materials and Methods

To better understand the performance of the textile–silicone composites, the specimens were prepared in 2- and 4-layer structures. Powernet fabrics were used, and the silicone materials were prepared with thicknesses of 0.5 mm, 1 mm and 2 mm, respectively. Their tensile strength and recovery, compression elasticity, impact resistance, thermal conductivity and water vapour permeability were measured and systematically compared with conventional silicone sheet and an inlaid spacer fabric.

### 2.1. Preparation of Materials

Two types of commercially available warp knitted powernet fabrics Fabric A (#34136) and Fabric B (#34471S) which are commonly used in clinical applications were sourced from Best Pacific Textile Ltd. (Hong Kong). The warp knitted structure of the fabrics imparts a high extensibility and excellent recovery behaviour in both wale and course directions [21,22] that not only allows free body movement and enhances wear comfort, but also assures dimensional stability upon prolonged use. These elastic properties provide an even pressure distribution to the human body, which is widely applied in pressure garments [23]. Protective equipment like wrist guards and knee braces are commonly used in sports to prevent hyperextension and injuries of joints [24]. The use of 4-way stretch powernet fabric is believed to provide additional support to the composite structure while maintaining wear comfort during posture changes, especially for joint rotational movement. Table 1 shows the fabric specifications, including the fibre contents and structure of the selected powernet fabrics. A two-component room temperature vulcanizing silicone (Silbione™ RTV-4410 A & B, Elkem Silicones, Rana, Norway) was used, which is a medical grade silicone rubber material with a Shore hardness of 10A and viscosity of 1600. It is typically used for the cushioning layer in custom-designed prostheses and orthoses during rehabilitation treatment.

### 2.2. Fabrication of Textile–Silicone Composite

**Figure 1:**
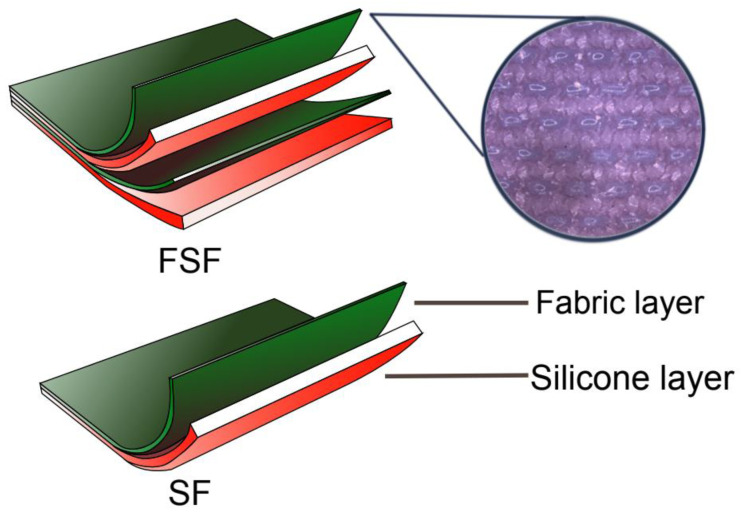
Illustration of textile–silicone composite structures: two-layer structure (SF) composed of one layer of silicone and powernet material, and four-layer structure (FSF)composed of two layers of silicone and powernet material, respectively. The image on the right shows the microstructure of laminated powernet in the textile–silicone composite.

Figure 1 shows the textile–silicone composite specimens, which are prepared as a two-layer structure (SF) and four-layer structure (FSF). SF was composed of a layer of silicone with a thickness of 1 mm or 2 mm, and a layer of powernet material. FSF has two powernet layers of which one is fully embedded inside the 0.5 mm or 1 mm silicone material. Hence, the specimens are prepared in 2- and 4-layer structures, respectively, while each layer of silicone material is prepared with a thickness of 0.5 mm, 1 mm or 2 mm, respectively. A total of eleven samples were prepared for the mechanical comparison (Table 2).

### 2.3. Experimental

The composite thickness was measured with a dial thickness gauge (Model H, Peacock OZAKI MFG. Co., Ltd., Tokyo, Japan) in accordance with ASTM D1777-96 Standard Test Method for Thickness of Textile Materials [25]. All of the material tests were conducted under a controlled temperature of 21 ± 1 °C and humidity of 65 ± 5%. The specimens were preconditioned for at least 24 h before the testing was carried out.

#### 2.3.1. Tensile Strength and Recovery

The tensile strength and recovery test was conducted with an Instron 4411 (Norwood, MA, USA) universal testing machine (with a load cell of 5 kN). Three specimens with dimensions of 32 mm (width) × 210 mm (length) of each sample were prepared and stretched repeatedly to 60% strain at a rate of 500 mm/min for eighty cycles to obtain a consistent stress–strain response [26]. The percentage recovery was also measured after eighty cycles. The tensile strength and recovery of the textile–silicone composites and inlaid spacer fabric were measured in both the wale and course directions.

#### 2.3.2. Compression Recovery

An Instron 5566 (Norwood, MA, USA) universal testing machine (with a load cell of 10 kN) was used for the compression test in accordance with ASTM D575-91 Standard Test Methods for Rubber Properties in Compression [27]. Three circular specimens with a diameter of 28.6 ± 0.1 mm were cut from the samples based on the testing standard in ASTM D575. The materials were tested at a maximum strain of 60% with a deflection rate of 40 mm/min for 10 cycles. The maximum compressive stress after compression and rate of recovery were then determined. The rate of recovery was calculated by the change in thickness as shown in Equation (1):

(1) $$\mathrm{Rate}\;\mathrm{of}\;\mathrm{recovery}=\left(1-\frac{h2-h1}{h1}\right)\times 100\%,$$

where *h_1_* is the thickness of the height of the sample before compression, while *h_2_* is the thickness after compression.

#### 2.3.3. Impact Force Reduction

A dropping test that followed the standard testing method of ASTM D2632-15 [28] was conducted to evaluate the impact resistance of the samples. Two specimens with dimensions of 100 mm × 100 mm were placed and stacked together before the test. During testing, a ball bearing was released from a height of 400 mm inside a vertical tube and allowed to fall onto the stacked samples and repeated ten times. The force of the impact was measured by using a load cell at the bottom of the apparatus. The impact force reduction capability was calculated as the percentage reduction in maximum force redacted when the ball struck the sample versus the direct force of the ball when dropped onto the ground surface. The equation of impact force reduction can be presented as below:

(2) $$FRx=\left(1-\left(\frac{Fx}{Fo}\right)\right)\times 100\%,$$

where *FRx* is the impact force reduction of the sample (%), *Fx* is the measured peak force for the sample (N) and *F*o is the measured peak force for the ground surface [29].

#### 2.3.4. Thermal Conductivity

The thermal conductivity of the samples was determined by using KES-F7 Thermo Labo (Kato Tech Co., Ltd., Kyoto, Japan). Three samples with dimensions of 10 cm × 10 cm were placed between a heat plate or the BT box (pressure at measurement 6 gf/cm^2^) at a temperature of 35 °C, which simulates skin temperature, and a water box at 25 °C to simulate the temperature of the external environment. The amount of heat conducted through the sample to the water box due to the difference in temperature was measured using the following equation:

(3) $$k=\frac{W\times D}{A\times \Delta T_{0}}\;\left(\frac{W}{\mathrm{cm}\cdot {}^{\circ}C}\right)$$

where *k* is the thermal conductivity (W/cm·°C), *W* = watts (W), *D* denotes the thickness of the sample (cm), *A* is the area of the heat plate or BT box (25 cm^2^) and Δ*T*_0_ represents the difference in temperature between the BT box and water box (i.e., 10 °C). The result is then converted and present in SI units with the following equation:

(4) $$k_{si}\;(W/m\cdot K)=k\times 10^{2}$$

#### 2.3.5. Water Vapour Permeability

The BS 7209:1990 Test Standard for Properties of Water Vapor Permeable Garment Fabrics [30] was used to determine the ability of water vapour to pass through the sample. Three specimens from each test sample were sealed over an open mouth test dish that contained water for 24 h under controlled conditions with a temperature of 20 ± 2 °C and humidity of 65 ± 2%. The water vapour permeability index was then calculated by using the weight difference of the assembly, which can be presented as follows:

(5) $$WVP=\frac{24\;M}{A\;t}$$

where *M* denotes the mass of the assembly (g) over a time period *t*, *t* is the time between the successive weighing of the assembly in hours and *A* represents the area of the exposed test specimen (m^2^).

### 2.4. Statistical Analysis

The five types of material properties of the eleven experimental samples were inputted into the Statistical Package for the Social Sciences (SPSS) Version 23.0 (IBM Corp., Armonk, New York, NY, USA) program for analysis. A multivariate analysis of variance (MANOVA) was used to examine the effects of three material factors, (1) structure, (2) thickness and (3) type of knitted fabric, on the properties of tensile stress and recovery, compression behaviour, impact force reduction, thermal conductivity and moisture permeability. The least significant difference (LSD)-adjusted post hoc tests were subsequently conducted to compare the main effects among the different samples. The significance level of the statistical analysis was set at *p* < 0.05.

## 3. Results and Discussion

### 3.1. Tensile Properties

#### 3.1.1. Tensile Strength

**Figure 2:**
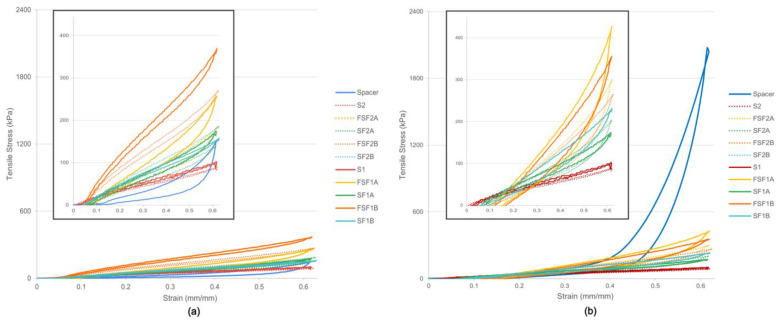
Stress–strain curves of samples (**a**) in wale direction after 80 cycles and (**b**) in course direction after 80 cycles.

A summary of the test results is presented in Table 3. The stress–strain curves in Figure 2 show that the tensile strength of the textile–silicone composites significantly increased in both wale and course directions compared to the silicone control samples (*p* < 0.05). The textile–silicone composites had increased tensile stress up to 315% as compared to the silicone control samples. Xu et al. [31] had similar findings in that the tensile strength of fabric rubber composites was enhanced by 69–386% with the reinforcement of different fabrics. As compared with the powernet fabric itself, Fabric A and Fabric B showed enhancements of up to 161.9% and 352.2% in maximum tensile load, respectively, when incorporated into a silicone layer. Although no significant differences were found between Fabrics A and B, the textile–silicone composites composed of Fabric A consistently exhibited higher maximum stress in the course direction as opposed to Fabric B. This aligns with the fabric properties shown in Table 4, where Fabric A has a much higher Young’s modulus and stiffness in the course direction than Fabric B because of the lower spandex content [32]. As indicated by Yin et al. [33], loop configuration can significantly influence the strain distributions where circular loops have a higher stress concentration factor (36%) than rectangular loops. The loop shape and density of Fabric A (with a width and length of 0.42 mm and 0.70 mm, respectively) result in a higher stress concentration factor compared to Fabric B (with a width and length of 0.31 mm and 0.67 mm, respectively), thus indicating a higher maximum stress.

Furthermore, the FSF textile–silicone composites were found to have significantly higher tensile stress (*p* < 0.05) than both the SF (*p* = 0.045 and 0.034) and control samples (*p* = 0.033 and 0.024) in both wale and course directions after 80 cycles of tensile testing, thus showing superior tensile strength. The findings suggest that the additional fabric layers exhibit the higher strength of the silicone rubber, which prevents the composite from breaking. Samples with an FSF structure, composed of two layers of powernet fabrics, hence resulted in the highest tensile strength.

On the other hand, the composites with 1 mm thick silicone were found to have a significant increase in tensile stress compared to the samples composed of only silicone (*p* = 0.041), corresponding to about a 30% increase in strength. This might indicate that a silicone matrix can provide stability for elasticity but not tensile strength. The thicker layer of silicone membrane might eliminate the mechanical properties of the fabric layer and lead to the reduction in strength of all of the samples.

As compared to the inlaid spacer sample, the textile–silicone composites have a slightly higher tensile strength in the wale direction. However, the spacer sample has a significantly higher tensile strength in the course direction than all of the other samples in terms of the fabric, structural and thickness parameters (*p* = 0.005). This is because the inlaid spacer sample is composed of two flat knitted face fabrics where the fibres inside are aligned and interlocked to form a cohesive structure that enhances its tensile strength by distributing the load across multiple fibres. As the silicone inlay is inserted in the course direction, the tensile stress is further enhanced, and the load is directly applied and restricted by the silicone tube. Unlike 2D structure, the 3D tubes have a much higher strength that prevents the spacer fabric from deformation in the course direction. On the other hand, silicone rubber is a homogeneous material which lacks fibre alignment and interlocking in its structure. However, after incorporating powernet fabric, the structure of the fabric limits the deformation of the silicone rubber, which therefore provides a more stable design in all directions, especially for the wale direction. Textile–silicone composite FSF, with higher stress, can be applied in prosthetic liners or knee braces, which require restriction in joint movement and muscle support [34]. The SF structure, with lower tensile stress, can be used for cushioning the bony prominence of amputees with enhanced comfort for daily usage.

#### 3.1.2. Tensile Elasticity and Recovery

No significant difference was identified with changes in thickness, fabric type and structure. Figure 2 shows the non-linear elastic behaviour of all of the samples after 80 cycles of tensile testing. The control and most of the textile–silicone composite samples exhibit good elasticity and can almost recover back to their original shape at the smallest strain of 0.15, where hysteresis started when the strain was increased to 0.3. In comparison to the textile–silicone composite samples, the control samples S1 (91.8%) and S2 (96.4%) showed a better performance in recovery in both the wale and course directions after 80 loading–unloading cycles. After adding fabric layers to the composite, the rate of recovery was reduced by around 9% in the composite samples with 1 mm and 2 mm thick silicone in the wale direction. The reduction is more obvious when tested in the course direction where the recovery rate was reduced by 22% and 17% for the composite samples with 1 mm and 2 mm thick silicone, respectively. It is believed that the loop of the knitted fabric extends and deforms when a higher tensile force is applied. The yarns fail to recover back to their original shape due to the high friction between fibres and yarns, which thus leads to a reduction in elasticity and recovery [15]. Furthermore, the mean recovery percentage of all of the composite samples with 1 mm thick silicone was 87.9%, which is lower than that of the composites with 2 mm thick silicone (mean recovery percentage 89.6%). The samples with a thicker silicone layer had greater elasticity. Unlike the tensile strength results, the thicker layer of silicone rubber can eliminate the effect of the fabric layer, which leads to an increase in elasticity. Moreover, the change in structure in both textile–silicone composites and control samples were stable over time, with a mean difference ranging from 0.07 to 2.07% and 0.08 to 0.79%, respectively. As a result, the additional fabric layer can provide extra tensile strength to prevent breakage, while the silicone matrix can stabilize the composite structure, which results in higher elasticity.

As compared to the spacer sample with silicone tube inlay for cushioning, no significant difference was identified in the wale or course direction. However, the inlaid spacer sample tested in the wale direction was observed to have a lower rate of recovery compared to the samples with silicone and the plotted curves exhibited a larger hysteresis between loading and unloading (Figure 2a). The tensile properties of spacer fabric are mainly attributed to the knitted structure of face fabrics, where the spacer yarns only move with the displacement of the outer fabric [35]. The head loops of the spacer yarns rotate during vertical extension or horizontal shortening of the outer fabric when load is applied. The damage to the spacer yarns leads to the reduction in bonding and thus reduction in tensile strength and recovery, and results in structural instability over time with a mean difference of 2.65%. However, the rate of recovery of the spacer sample in the course direction is much higher (Figure 2b). This phenomenon is due to the restriction from the silicone tubes. Therefore, the deformation is eliminated with only a slight change in the structure.

### 3.2. Compression Behaviour

**Figure 3:**
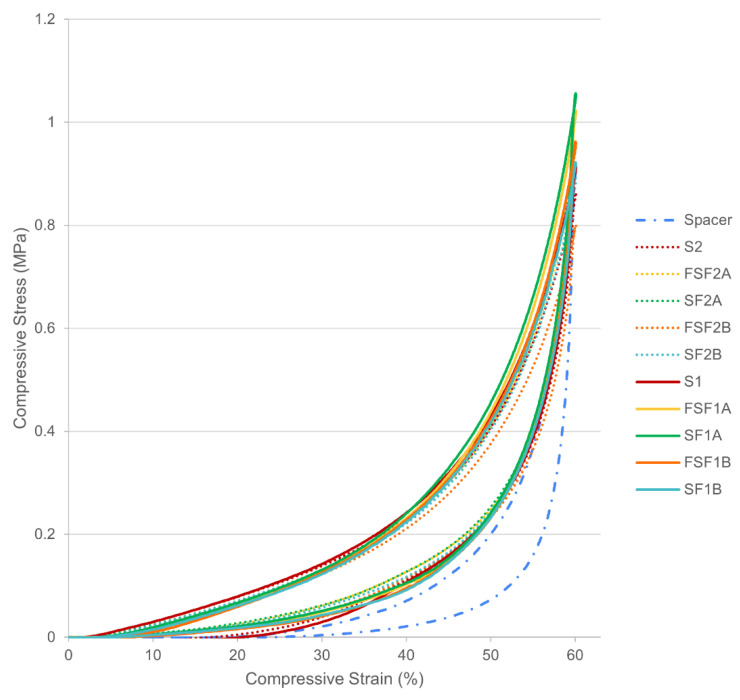
Stress–strain curves of spacer fabric, textile–silicone composites and control samples at the 10th cycle.

**Figure 4:**
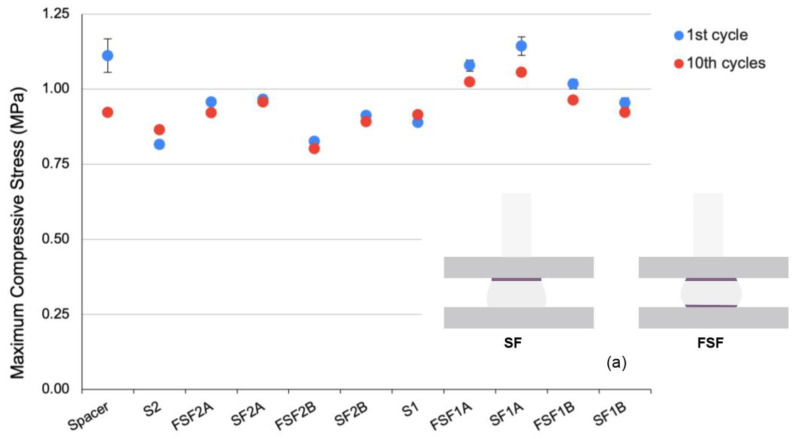
Maximum compressive stress of spacer fabric, textile–silicone composites and control samples: (**a**) illustration of deformation of textile–silicone composite with SF and FSF structures.

No significant difference was found among all of the samples for the maximum compressive stress in terms of different structures, thickness and types of fabrics. With reference to the stress and strain curves in Figure 3, the compression properties of all of the samples are observed to be non-linear. The textile–silicone composites have a higher maximum compressive stress and require higher forces than the control samples to be compressed to the same strain, especially SF1A, which increased by 16.5% (Figure 4). The textile–silicone structures increase the overall strength and stiffness of the composites and prevent the deformation of the silicone rubber when subjected to compression. Fabric A, which has a larger Young’s modulus, significantly increases the stiffness of the silicone rubber as compared to Fabric B. This shows that the fabric properties can affect the compression behaviour of the silicone composites. In contrast to the results for the tensile force, the two-layer structure SF was recorded to have a higher maximum compressive stress. Fabric adhered to one side of the silicone provides reinforcement and limits the deformation of the silicone on that particular side of the composite (Figure 4a). In contrast, when fabric is adhered to the upper and lower surfaces of the silicone, both sides of the silicone are evenly reinforced, which results in energy accumulation in the middle layer, and thus, lower overall compressive stress [36]. 

In terms of compression recovery, significant differences were only identified between the textile–silicone composites and spacer samples (*p* = 0.003) after ten cycles of compression. Although the inlaid spacer sample was observed to have the highest compressive stress after the first cycle of compression, a significant reduction was observed after repeated testing (Figure 3). In comparison to the inlaid spacer sample, the textile–silicone composite samples, especially those with 2 mm thick silicone, demonstrate more consistent compression behaviour, even after repeated testing, and can almost fully revert back to their original shape after ten cycles of compression (rate of recovery > 99%). However, the spacer sample was unable to fully revert back to its original state (rate of recovery of 83%) and exhibited greater deformation at higher strain. This phenomenon can be explained by its three-dimensional structure, where the monofilament yarns started to deform and buckle when the stress exceeded a certain level and it could no longer maintain the connective structure, which led to the shearing of the fabric layers and yarn rotation [37]. A slight increase in stress can easily lead to the collapse and deformation of spacer fabric.

With a high recovery rate and lower compressive stress, the 2 mm textile–silicone composites are more applicable for padded bike gloves that require thicker and softer material properties to cushion the palm and reduce pressure points to support prolonged rides. The 1 mm textile–silicone composites that are thinner and harder can help people better withstand pain than thick and soft materials, and are more applicable for impact protective usages, just like the hand guard gloves that are often made of rigid materials to protect the bones and joints from fractures and injuries [38].

### 3.3. Impact Force Reduction

**Figure 5:**
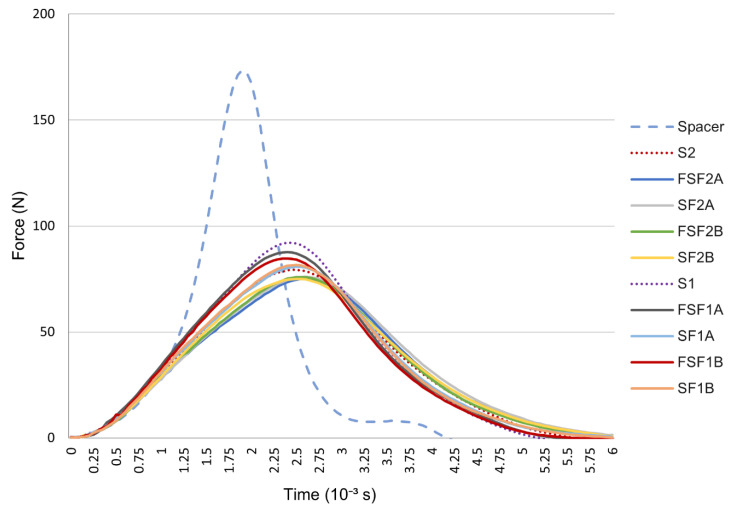
Force−time diagram of spacer fabric, textile−silicone composites and control samples.

Although no significant difference was observed among the samples in their force absorption performance in terms of structure, thickness and fabrics, all of the textile–silicone composites and control samples show a higher percentage of impact force reduction than the spacer sample, with the largest differences being 43% and 41%, respectively, which indicates a better performance in impact absorption. This is in line with the results of Maurel et al. [39], who modified a commercially available padded wrist guard by adding an additional gel layer, which led to an approximate 50% reduction in impact force. As shown in the force–time diagram in Figure 5, the inlaid spacer sample has the highest peak force in a relatively short period of time from absorbing the high impact force imposed by the ball bearing, whereas the textile–silicone composites and control samples with a silicone layer of 2 mm in thickness, either with SF or FSF structures, have the lowest peak force with a relatively long period of reaction time for force absorption. The reaction time required for the impact force absorption is similar among the textile–silicone composites and control samples. The extended time intervals indicate that the control samples and the textile–silicone composites have gradually absorbed the force and acted as an energy buffer against impact forces [40]. As is already known, silicone rubber has excellent elasticity and flexibility, which allows this material to absorb and dissipate impact forces effectively over large areas to reduce the transmittance of peak forces to the object. On the other hand, spacer fabric is a 3D textile, so its ability to absorb impact forces mainly depends on factors like the density of spacer threads and inlaid materials. Inlaid materials placed in an appropriate pattern with an adequate density can effectively absorb and decrease the impact forces for a satisfactory effect [41]. However, spacer fabric that incorporates silicone tubes as the inlaid material might not be able to evenly distribute and cushion or dissipate energy as compared to a pure silicone or silicone composite absorptive layer. Compared to the silicone samples, the incorporation of powernet fabric slightly enhances energy absorption. According to Auroy et al. [42], the incorporation of a layer of glass fibre cloth reduces the shock-absorption capacity of silicone rubber, where the greater the hardness, the lower the shock-absorption capacity of silicone rubber. By using more elastic or resilient reinforced materials, the absorption capacity of silicone rubber can be increased. However, the effect of fabric was eliminated with the increase in silicone thickness. In terms of silicone thickness, the samples with 2 mm thick silicone have better shock-absorption behaviour regardless of whether the fabric is taken into consideration. With thicker material, more energy can be absorbed [43]. Therefore, a thicker absorptive layer has a higher force reduction capability.

### 3.4. Thermal Conductivity

According to Table 3, there are observable, but no significant, differences among all of the samples in terms of different structures, thickness and fabrics. The effects of Fabric A or B on the thermal conductivity of the SF structure with both 1 mm and 2 mm thick silicone were similar when compared to the corresponding control samples. The effects of the FSF structure were slightly higher with Fabric B (0.28 W/m·K) as compared to Fabric A as fabric backing (0.22 W/m·K). Similar results can also be observed in the textile-composite samples with an FSF structure and 1 mm thick silicone. The proposed textile–silicone composites in this study have higher thermal conductivity than commercial silicone liners (0.16 ± 0.02 W/m·K), as mentioned in the study of Cagle et al., which can conduct heat away from a warm limb to the cooler environment more efficiently [29]. As an inorganic synthetic polymer, typical silicone rubber has a very low intrinsic thermal conductivity of around 0.2 to 0.5 W/m·K [44]. Thermal conductivity can be increased by incorporating more efficient thermal conductive fillers [33]. Fabric A, which is less conductive than Fabric B according to Table 4, inhibits heat transmission through the composite. It is believed that fabric inserted into a structure can affect the thermal behaviour of the composite structure aside from adhering the fabric to the top of the composite. Apart from conductive fillers, incorporating fabric can also enhance the thermal conductivity of composites, in which the higher the content or more conductive the material, the higher the conductivity. On the other hand, the incorporation of silicone has increased the thermal conductivity of powernet fabrics itself up to 310.7% (Fabric A) and 330.8% (Fabric B), respectively, which can improve the cooling effect of pressure garments.

Nevertheless, all of the textile-composite samples are more conductive than the spacer sample, which is due to the presence of air pockets within the spacer fabric structure that enhance its insulating properties; on the other hand, this inhibits its heat transfer performance.

### 3.5. Water Vapour Permeability

Without perforation, the textile-composites have a significantly lower water vapor permeability than the spacer sample in terms of structure, thickness and fabric (*p* = 0.010) (Table 3). The knitted loops of the fabric layers allow water vapour to pass through and prevent the trapping of moisture. No significant difference was found between the textile- composites and control samples, as the holes of the powernet were blocked by an impermeable silicone layer, consistent with the result of previous research [45]. However, there was a noticeable trend that the samples with the two-layer SF structure were more water vapour permeable than those with the four-layer FSF structure, and thinner samples also showed a better performance. This might be because the water vapour can more readily escape from the micro-pores of the thinner film or unexpected voids and evaporate.

## 4. Conclusions

In this study, a series of textile–silicone materials were developed with different structures, fabric and thickness to systematically evaluate their cushioning performance, mechanical and thermal properties, and to compare them with a conventional silicone cushioning sheet and spacer fabric with silicone inlay. The results show that the textile–silicone composites have superior tensile strength which can be improved up to 300% (FSF1A) as compared with pure silicone elastomer. Composites made of two layers of powernet fabric (FSF) exhibit higher tensile strength and slight improvement in compression recovery than those made of one layer of fabric; it is believed that the powernet fabric layer can improve the structural stability to prevent the silicone composite from breakage or deformation, which supports the hypothesis of this study. Nevertheless, the influence of the textile–silicone structure on force reduction, thermal conductivity and water vapour permeability is not apparent. Despite this, as compared to the control sample (pure silicone), the textile–silicone structure not only preserves the cushioning effect, but also increases thermal conductivity. However, the immersion of silicone unexpectedly eliminated the advantages of the textile fabric in terms of permeability. Even though the inlaid spacer fabric is highly air and water vapour permeable, its mechanical properties for tensile and compression recovery, impact force reduction and thermal conductivity fall short of the silicone-based samples. The findings from this study show the advantages of textile–silicone composites in terms of mechanical properties, especially for tensile and impact reduction, while at the same time disclosing the limitations of its impermeability. Future study should focus more on improving the porosity and permeability of textile–silicone composites in order to fill the demand for functional and comfortable protective and cushioning apparel products in healthcare and/or sports applications.

## Figures and Tables

**Table 1:** Fabric specification.

Fabric	Fibre Contents	Thickness (mm)	Weight (g/m^2^)	Gauge and Yarn Density (Threads/cm)	Knitting Structure	
					Face	Back
A	83% Polyamide, 17% Elastane	0.40	165	28 gauge, 14/11	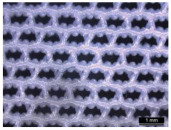	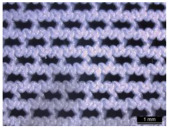
B	62% Polyamide, 38% Elastane	0.39	195	28 gauge, 17/13	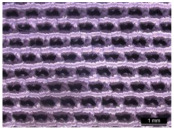	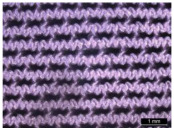

**Table 2:** Specifications of tested samples.

Sample	Thickness	Weight (g/cm^2^)	Structure	Cross-Sectional Image
Textile–silicone composites with 2 mm thick silicone				
SF 2A	2.38 mm	0.32 g	1 layer of Fabric A and 1 layer of 2 mm thick silicone	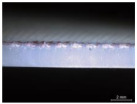
FSF 2A	2.54 mm	0.38 g	2 layers of Fabric A and 2 layers of 1 mm thick silicone	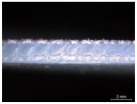
SF 2B	2.42 mm	0.31 g	1 layer of Fabric B and 1 layer of 2 mm thick silicone	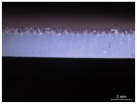
FSF 2B	2.60 mm	0.33 g	2 layers of Fabric B and 2 layers of 1 mm thick silicone	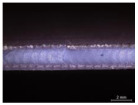
Textile–silicone composites with 1 mm thick silicone				
SF 1A	1.46 mm	0.19 g	1 layer of Fabric A and 1 layer of 1 mm thick silicone	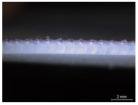
FSF 1A	1.56 mm	0.20 g	2 layers of Fabric A and 2 layers of 0.5 mm thick silicone	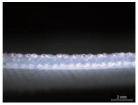
SF 1B	1.44 mm	0.18 g	1 layer of Fabric B and 1 layer of 1 mm thick silicone	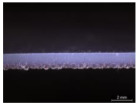
FSF 1B	1.52 mm	0.19 g	2 layers of Fabric B and 2 layers of 0.5 mm thick silicone	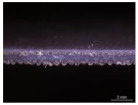
Inlaid spacer structure and silicone as the control				
SPACER	4.20 mm	0.16 g	Spacer fabric with silicone tube inlay	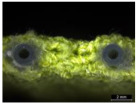
S1	0.96 mm	0.12 g	Silicone only (control)	
S2	2.04 mm	0.26 g	Silicone only (control)	

**Table 3:** Mechanical properties of the samples tested.

Sample		Maximum Tensile Stress after 80 Cycles (kPa)		Tensile Recovery (%)		Compressive Stress after 10 Cycles (MPa)	Compression Recovery (%)	Force Reduction (%)	Thermal Conductivity (W/m.K)	WVP (g/m^2^/day)
		Wale	Course	Wale	Course					
FSF	FSF2A	186.46	299.05	88.2	79.7	0.92	100.0	81.65	0.22	14.78
	(SD)	(3.196)	(2.055)	(2.367)	(0.832)	(0.002))	(0.000)	(0.914)	(0.015)	(1.308)
	FSF1A	257.07	427.42	83.4	74.8	1.02	100.0	78.45	0.18	22.18
	(SD)	(3.550)	(2.001)	(1.379)	(0.797)	(0.003)	(0.006)	(0.596)	(0.033)	(1.301)
	FSF2B	270.77	266.21	91.6	82.2	0.80	99.7	82.06	0.28	16.63
	(SD)	(4.787)	(3.670)	(2.278)	(0.516)	(0.004)	(0.006)	(1.620)	(0.059)	(1.308)
	FSF1B	369.66	355.55	89.5	72.0	0.96	99.6	79.09	0.23	22.18
	(SD)	(4.236)	(3.804)	(1.084)	(1.820)	(0.003))	(0.007)	(1.525)	(0.087)	(3.924)
SF	SF2A	139.37	203.59	87.7	86.3	0.96	100.0	81.33	0.23	16.63
	(SD)	(1.138)	(5.035)	(0.585)	(0.920)	(0.003)	(0.000)	(1.661)	(0.036)	(1.308)
	SF1A	173.23	174.25	87.0	86.6	1.06	99.6	80.12	0.20	25.87
	(SD)	(4.165)	(2.828)	(1.760)	(0.556)	(0.004)	(0.129)	(0.902)	(0.015)	(2.609)
	SF2B	186.57	170.39	90.8	87.4	0.89	99.0	81.40	0.23	18.48
	(SD)	(3.741)	(5.473)	(1.473)	(0.622)	(0.003)	(0.172)	(0.988)	(0.107)	(1.308)
	SF1B	157.67	231.17	91.5	81.7	0.92	99.6	80.00	0.21	29.57
	(SD)	(5.716)	(3.274)	(0.443)	(2.716)	(0.002)	(0.063)	(1.102)	(0.055)	(2.616)
2 mm thick silicone	S2	87.97		96.4		0.86	100.0	80.69	0.24	18.48
	(SD)	(1.172)		(0.916)		(0.002)	(0.000)	(1.505)	(0.071)	(1.308)
1 mm thick silicone	S1	103.02		91.8		0.91	99.9	77.34	0.21	31.42
	(SD)	(5.056)		(1.058)		(0.008)	(0.069)	(1.535)	(0.226)	(3.924)
Spacer fabric	Spacer	152.44	1727.60	69.9		0.92	83.0	57.39	0.13	698.54
	(SD)	(0.700)	(176.176)	(5.013)		(0.005)	(2.410)	(5.336)	(0.233)	(2.609)

**Table 4:** Physical properties of the powernet fabrics.

Fabric	Young’s Modulus		Poisson’s Ratio		Thermal Conductivity (W/m·K)	WVP (g/m^2^/day)
	Wale	Course	Wale	Course		
A	0.631	1.259	0.193	0.253	0.056	844.5
B	0.548	0.519	0.123	0.023	0.065	834.4

## Data Availability

Data are contained within the article.
